# Tickborne Arbovirus Surveillance in Market Livestock, Nairobi, Kenya

**DOI:** 10.3201/eid1207.060253

**Published:** 2006-07

**Authors:** Rosemary Sang, Clayton Onyango, John Gachoya, Ernest Mabinda, Samson Konongoi, Victor Ofula, Lee Dunster, Fred Okoth, Rodney Coldren, Robert Tesh, Amelia Travassos da Rosa, Stacy Finkbeiner, David Wang, Mary Crabtree, Barry Miller

**Affiliations:** * Kenya Medical Research Institute, Nairobi, Kenya;; † United States Army Medical Research Unit, Nairobi, Kenya;; ‡ University of Texas Medical Branch, Galveston, Texas, USA;; § Washington University School of Medicine, Saint Louis, Missouri, USA;; ¶ Centers for Disease Control and Prevention, Fort Collins, Colorado, USA

**Keywords:** arbovirus, tickborne virus, Dugbe virus, Dhori virus, Thogoto virus, Kadam virus, Bhanja virus, Foot-and-mouth disease virus, Nairovirus, surveillance, research

## Abstract

To identify tickborne viruses circulating in Kenya and the surrounding region, we conducted surveillance at abattoirs in Nairobi, Kenya. Species of ticks collected included *Rhipicephalus pulchellus* (56%), *Amblyomma gemma* (14%), *R. appendiculatus* (8%), *A. variegatum* (6%), and others. A total of 56 virus isolates were obtained, 26 from *A. gemma*, 17 from *R. pulchellus*, 6 from *A. variegatum*, and 7 from other species. Virus isolates included Dugbe virus (DUGV), an unknown virus related to DUGV, Thogoto, Bhanja, Kadam, Dhori, Barur, and foot-and-mouth disease (FMDV) viruses. This is the first report of Dhori virus isolation in East Africa and the first known isolation of FMDV associated with tick collection. Our results demonstrate the potential for tickborne dissemination of endemic and emergent viruses and the relevance of *A. gemma* in the maintenance of tickborne viruses in this region.

## Abstract 2

Numerous tickborne viruses, including Dhori virus and foot-and-mouth disease virus, were isolated.

Viruses transmitted by blood-feeding arthropods (arboviruses) are responsible for some of the most serious emerging infectious disease problems facing the world today. Arthropodborne viruses constitute the largest biologic group of vertebrate viruses. Their considerable number and diversity suggest that arthropod vector transmission offers distinct survival benefits for the virus. Approximately 50% of arbovirus isolations from field-collected arthropods are from mosquitoes, and 25% are from ticks; however, this difference may represent a sampling bias, since many more mosquitoes are collected and tested for virus than ticks. To investigate the abundance of tickborne arboviruses in Kenya and the surrounding region, we collected and tested ticks infesting livestock driven to market at 2 major abattoirs in Nairobi, Kenya. These abattoirs receive the bulk of animals slaughtered for Nairobi and its environs, which is the largest livestock market in the country. Approximately 30% of animals slaughtered in these abattoirs come from within Kenya; the rest are from neighboring countries, including Ethiopia, Sudan, Somalia, and Tanzania.

Among pastoral communities in this region, livestock are frequently maintained in enclosures close to human habitation, and small ruminants sometimes sleep inside houses overnight for security reasons. Such practices increase the potential for zoonotic virus transmission between animals and humans. Poor husbandry and grazing practices put great pressure on land resources, which results in the need to continuously move large numbers of animals, especially cattle, in search of pasture. In some parts of East Africa, these pastoral communities exist near wildlife parks, and wildlife and livestock sometimes mix, which allows transfer of ticks and possibly viruses between these animal groups. Additionally, livestock marketing practices allow movement of animals across borders in the region, which allows ticks and tickborne viruses to move between countries.

Previous surveillance reports based on virus isolations or serologic studies in cattle from Kenya, the Central African Republic, and South Africa have identified tickborne arboviruses from the *Bunyaviridae*, *Flaviviridae*, *Rhabdoviridae*, *Reoviridae*, and *Orthomyxoviridae* (*1**–**4*). The genus *Nairovirus*, family *Bunyaviridae*, includes 37 named viruses that are principally tickborne (*5**–**7*). The most serious human pathogen among the tickborne viruses in the African region is Crimean-Congo hemorrhagic fever virus (CCHFV), a member of the *Nairovirus* genus that can cause fatal hemorrhagic disease (*8**,**9*). Outbreaks of Crimean-Congo hemorrhagic fever have occurred in People's Republic of China, South Africa, Pakistan, and Russia (*10**–**12*). The first reported human case of this disease in Kenya occurred recently at a farm that was heavily infested by ticks (*13*). Nairobi sheep disease virus (NSDV), also in the genus *Nairovirus*, causes fever, hemorrhagic gastroenteritis, and abortion in sheep and goats in East Africa (*14*). Epizootics of NSDV have been reported in parts of Africa where susceptible herds of sheep have been moved to NSDV-endemic areas, resulting in decimation of whole herds (*14*). Dugbe virus (DUGV), another member of the *Nairovirus* genus, has been repeatedly confirmed in tickborne virus surveys in Africa and causes febrile illness and thrombocytopenia in humans (*2*). Bhanja virus (BHAV), an unassigned member of the *Bunyaviridae* family, has also been isolated in this region (*1**,**2*). Other tickborne viruses present in Africa include Thogoto virus (THOV) (genus *Thogotovirus*, family *Orthomyxoviridae*), isolated in Kenya; Barur virus (family *Rhabdoviridae*), isolated in Somalia; and Kadam virus (KADV) (genus *Flavivirus*, family *Flaviviridae*) and Chenuda virus (genus *Orbivirus*, family *Reoviridae*), confirmed serologically in cattle in South Africa (*1**,**2**,**4*).

Our study isolated and identified 6 previously known tickborne arboviruses, including DUGV, BHAV, THOV, Dhori virus (DHOV), KADV, and Barur virus. In addition, 2 viruses related to DUGV were isolated. An unexpected result of this study was the isolation of foot-and-mouth disease virus (FMDV) from tick pools.

## Materials and Methods

### Tick Collection and Processing

Ticks were collected from the hides of flayed animals between September and November 1999 at the Njiru and Dagoretti abattoirs, on the outskirts of Nairobi, Kenya. Attached ticks were pulled off manually and placed in sterile plastic vials, which were loosely capped and transported to the laboratory. The origin of individual sampled animals could not be determined. All animals to be slaughtered for the day were put in 1 enclosure, irrespective of origin.

In the laboratory, ticks were washed twice with sterile water to remove excess particulate contamination from animal hides, rinsed once with 70% ethanol, and then rinsed twice with minimum essential medium (MEM), with antimicrobial agents (100 U/mL penicillin, 100 μg/mL streptomycin, and 1 μL/mL amphotericin B). Ticks were identified by sex and species by using appropriate identification keys (*15**,**16*), transferred to sterile vials, and stored at –80°C until homogenized for virus isolation. Voucher specimens were prepared in ethanol, and identifications were reviewed at the International Centre of Insect Physiology and Ecology, Nairobi. Ticks were later thawed at room temperature, identifications were confirmed, and ticks were pooled into groups of 2 to 50, depending on the size of the ticks and according to species, collection dates, and sites. Each pool was homogenized by using 90-mesh alundum in a prechilled, sterile mortar and pestle with 1.6–2 mL ice cold bovine albumin (BA)-1 medium (1× medium 199 with Earle's salts, 1% BA, 100 U/mL penicillin, 100 μg/mL streptomycin, and 1 μL/mL amphotericin B). The homogenates were clarified by low-speed centrifugation at 1,500 rpm for 15 minutes at 4°C, and supernatants were aliquoted and stored at –80°C. In the case of *Hyalomma* species, the primary vectors of CCHFV, each pool was screened by reverse transcription–polymerase chain reaction (RT-PCR) for CCHFV before tissue culture injection was conducted (Table 1).

**Table 1:** Reverse transcription–polymerase chain reaction primers used for arbovirus isolation, Nairobi, Kenya

Virus target	Target gene	Primer designation	Primer sequences*
Crimean-Congo hemorrhagic fever	N	CCHF F2	TGGACACCTTCACAAACTC
		CCHF R3	GACAAATTCCCTGCACCA
Dugbe	N	DG S1	TCTCAAAGACAAACGTGCCGCAG
		DG S5	TGCAACAACTGGATGTGTGA
Nairobi sheep disease	N	NSD 12f	GAATGGTCGAACGTGGAC
		NSD 16r	TGCTGTCAGGACACCAGG
Thogoto	N	THO NF	CCTGCAGGGGCGGAAGTTATG
		THO NR	AAAATCCTCGCAGTTGGCTATCA
Flaviviruses	NS5	FLAVI fu2	GCTGATGACACCGCCGGCTGGGACAC
		FLAVI cfd3	AGCATGTCTTCCGTGGTCATCCA
Bunyamwera and California serogroups	N	BCS82C	ATGACTGAGTTGGAGTTTCATGATGTCGC
		BCS332V	TGTTCCTGTTGCCAGGAAAAT
Rift Valley fever	G2	RVF-M-727f	GGAACCCCTGCATGAAAGAGA
		RVF-M-1565r	CGATCCTGTGACGCAAACTC
Dhori	N	DHO NF2	TGGTACCCTTTTCTTGCTTCACTCC
		DHO NR2	TGCTCTTCCTCGGCTCAAACACCA
Babanki	C/E3	BAB 1007f	TGGCCATGGAAGGTAAGGTAAT
		BAB 1569r	TATGGCGTTGAGCAGGGTATC
Hazara	N	HAZ 803f	CTGGTTGAGCTAGAGGGGAAAGACG
		HAZ 1304r	GGGCGGCATCATCGGGACTG
Coxsackie B4	Pol	COX 6749f	ACTTTGTGAGAGGGGGTATGC
		COX 7151r	ACGTGGTATTGGGTGTTTTT
Koutango	NS5	KOU 176f	TCAGGGAGGTGGGAGGTAAAC
		KOU 734r	TCATGCCATCCAACAGAAGGT
Saboya	NS5	SAB 226f	GCAGGCTGGGACACAAAGAT
		SAB 815r	CTACAAGGGGCAATGATGGTTC
Ndelle	l3	NDE 655f	GGGGTTTTCTGGCTAATGTCAC
		NDE 1238r	GGGCCTGTCCAGTCTTTTTG
Middleburg	E2	MID 1939f	TACATGCCCCGAAGGTGACT
		MID 2458r	CGGGATGGTGTTCGGTAAAG

*Primer sequences 5´–3´.

### Virus Isolation

For virus isolation in cell culture, Vero cells were grown in 25-cm^2^ cell culture flasks to 80% confluency in MEM with 10% fetal bovine serum (FBS), 2% glutamine, 100 U/mL penicillin, 100 μg/mL streptomycin, and 1 μL/mL amphotericin B. Cells were rinsed with sterile saline, and 0.2 mL clarified tick homogenate was added followed by injection at 37°C for 45 minutes to allow virus adsorption. After incubation, cells were rinsed with saline and maintenance medium (MEM with Earle's salts, with 5% FBS, 2% glutamine, 100 U/mL penicillin, 100 μg/mL streptomycin, and 1 μL/mL amphotericin B) was added. Cells were incubated at 37°C and observed daily for signs of cytopathic effects (CPE). The pooled infection rate program (PooledInfRat, Centers for Disease Control and Prevention, Fort Collins, CO, USA; http://www.cdc.gov/ncidod/dvbid/westnile/software.htm) was used to compare virus infection rates in the tick species collected and processed in this study.

### Virus Identification

Agents causing CPE in tissue culture were initially identified to virus group by using the indirect immunofluorescent antibody assay (IFA) on spot slides of infected Vero cells with polyvalent mouse hyperimmune ascitic fluids obtained from the National Institutes of Health Reference Reagents Program. Fluorescein isothiocyanate–conjugated goat-anti-mouse immunoglobulin G was the secondary antibody.

RT-PCR was also used to identify most of the virus isolates obtained from tissue culture. RNA was extracted from cell culture supernatants with the QIAamp Viral RNA kit (Qiagen, Valencia, CA, USA) according to the manufacturer's recommended protocol. RT-PCR was performed with the Titan One Tube RT-PCR kit (Roche, Indianapolis, IN, USA) with primers mainly targeting the known African tickborne viruses (Table 1). References are available from the authors for previously published primers. All other primers were designed for this study to amplify a specific fragment from the virus listed and have not been tested for cross-reactivity with other related or unrelated viruses. RT-PCR was also performed on RNA extracted from uninfected Vero cells as a negative control. Amplified DNA fragments were visualized by electrophoresis on 0.8%–1.0% agarose gels. DNA fragments were extracted from gels with the QIAquick Gel Extraction Kit (Qiagen), and DNA was eluted in 20 μL 10 mmol/L Tris-HCl, pH 8.5, and stored at –20°C. RT-PCR fragments were sequenced with the CEQ DCTS Quick Start kit (Beckman Coulter, Inc., Fullerton, CA, USA) with listed primers and analyzed with a CEQ 8000 automated sequencer (Beckman Coulter, Inc.). Both strands of DNA were sequenced. Nucleic acid sequences were compared with the GenBank database by using the BLAST program (http://www.ncbi.nlm.nih.gov/BLAST).

Additional methods used to identify selected isolates included complement fixation (CF) and hemagglutination-inhibition tests (*17*) and panviral microarray-based genotyping (*18*). Alignment of nucleic acid and deduced protein sequences was conducted by using the MegAlign program (Lasergene 6.1, DNASTAR, Inc., Madison, WI, USA); phylogenetic and molecular evolutionary analyses were conducted with MEGA version 3.0 (http://www.megasoftware.net).

## Results

### Tick Collection

A total of 15,851 ticks were collected and processed in 1,071 pools for virus isolation. Species of ticks collected and their proportions in the collection are shown in Table 2. The predominant species collected was *Rhipicephalus pulchellus* (56%), followed by *Amblyomma gemma*, *R. appendiculatus*, and *A. variegatum*. Other species were sampled in smaller numbers. However, the calculated virus pooled infection rate was higher for *A. gemma* than for *R. pulchellus* (Table 2). The number of *Hyalomma* specimens collected was comparatively small (3%). These species are the primary vectors of CCHFV; this agent was not among the viruses isolated.

**Table 2:** Tick species collected and their virus yield, Kenya*

Species	No. collected	No. virus isolates	Pooled infection rate
*Amblyomma variegatum*	994	6	6.16
*A. gemma*	2,160	26	11.05
*A. lepidum*	963	4	4.24
*A. coharens*	4	0	0
*Boophilus decoloratus*	985	1	1.01
*Hyalomma truncatum*	270	0	0
*H. albiparmatum*	112	0	0
*H. rufipes*	96	0	0
*H. dromedarii*	1	0	0
*Rhipicephalus appendiculatus*	1,228	2	1.63
*R. evertsi*	146	0	0
*R. pulchellus*	8,892	17	2.48
Total	15,851	56	

*DUGV, Dugbe virus; BHAV, Bhanja virus; THOV, Thogoto virus; DHOV, Dhori virus; KADV, Kadam virus; BARV, Barur virus, FMDV, foot-and-mouth disease virus.

### Virus Isolation and Identification

A total of 56 virus isolates were obtained from 51 tick pools; 52 of the 56 viruses were identified (Table 3). Five pools contained 2 different viruses. All of the isolated viruses caused CPE in Vero cells. The observed onset of CPE was 4–10 days postinfection. In the initial identification by IFA, 6 isolates reacted positively with the Thogoto group–specific antiserum (polyvalent 4), 33 isolates reacted with the Congo group–specific antiserum, 1 isolate reacted with the flavivirus group–specific antiserum (group B), and 1 isolate reacted with antiserum that included specificity to DHOV (polyvalent 10).

**Table 3:** Virus isolates obtained from ticks collected in Nairobi, Kenya

Species	Virus isolates									
	DUGV	DUGV-like	BHAV	THOV	DHOV	KADV	BARV-like	FMDV	Unknown	Total
*Amblyomma variegatum*	6	0	0	0	0	0	0	0	0	6
*A. gemma*	16	5	0	4	0	0	0	0	1	26
*A. lepidum*	2	1	0	1	0	0	0	0	0	4
*A. coharens*	0	0	0	0	0	0	0	0	0	0
*Boophilus decoloratus*	0	0	1	0	0	0	0	0	0	1
*Hyalomma. truncatum*	0	0	0	0	0	0	0	0	0	0
*H. albiparmatum*	0	0	0	0	0	0	0	0	0	0
*H. rufipes*	0	0	0	0	0	0	0	0	0	0
*H. dromedarii*	0	0	0	0	0	0	0	0	0	0
*Rhipicephalus appendiculatus*	0	0	1	0	0	1	0	0	0	2
*R. evertsi*	0	0	0	0	0	0	0	0	0	0
*R. pulchellus*	2	1	0	1	1	0	6	3	3	17
Total	26	7	2	6	1	1	6	3	4	56

Forty-five virus isolates were identified by using RT-PCR and nucleic acid sequencing with primers specific to known tickborne viruses or by CF assay or microarray-based genotyping. The identified isolates included 26 DUGV, 6 THOV, 6 Barur virus, 3 FMDV, 2 BHAV, 1 DHOV, and 1 KADV. DUGV was isolated most frequently (46%). Most DUGV isolates were recovered from *A. gemma* (62%), whereas the most commonly sampled tick, *R. pulchellus*, yielded only 2 DUGV isolates (8%) (Table 3).

Two of the virus isolates that were IFA-positive with Congo group antiserum were RT-PCR negative when primers specific for DUGV, CCHFV, Hazara virus, or BHAV, all of which were represented in the antiserum, were used. However, RT-PCR using NSDV nucleocapsid–specific primers and RNA extracted from these isolates produced 3 major bands, including one ≈880 bp in size; the expected band size for the NSDV-specific fragment was 887 bp. The 880-bp fragment was sequenced, and an alignment of 513 nt (nt) of this sequence with nucleocapsid sequences from DUGV, CCHFV, and NSDV showed 71%, 58%, and 60% homology, respectively, which suggests that these isolates were most closely related to DUGV. Alignment of the sequences from the 2 isolates showed them to be 95% homolgous. Specific primers were designed for this DUGV-like virus from sequence of the fragment described above. RT-PCR conducted with these primers produced bands of correct size and sequence with RNA from the DUGV-like virus isolates, while RT-PCR results using these primers with RNA from DUGV and BHAV were negative. RT-PCR and sequencing of all of the virus isolates using the primers designed from the DUGV-like virus sequence showed 4 additional isolates of this DUGV-like virus; 2 were from pools that also contained DUGV. Sequence homology between all 6 DUGV-like isolates was 95%–100%, based on a 508-nt alignment of the S segment of the virus RNA. Of the 6 isolates of this virus, 5 (83%) were from *A. gemma* pools, and 1 (17%) was from a pool of *R. pulchellus.* One additional isolate, obtained from a pool of *A. lepidum*, was identified as being DUGV-like by CF test. RT-PCR conducted on RNA extracted from this isolate with the primers designed for the DUGV-like virus described above produced a weak band. Sequence of this product was ≈80% homologous to the other isolates of DUGV-like virus and 70%, 63%, 57%, and 55% homologous to DUGV, NSDV, CCHFV, and Hazara virus, respectively, which suggests that it may be a different DUGV-like isolate.

Six isolates of THOV were obtained: 4 from pools of *A. gemma*, l from *A. lepidum*, and 1 from *R. pulchellus*. Since the THOV genome is segmented, a portion of each of the 6 genome segments from each isolate was sequenced and compared with available sequence from other THOV isolates to determine if reassortment of virus genome segments had occurred. No evidence was found for reassortment of virus segments. Phylogenetic analysis showed that the THOV isolates were most closely related to other African isolates from Uganda (1996), Kenya (1960), and Nigeria (1969) (data not shown).

The single isolate of DHOV, another member of the tickborne orthomyxovirus group, was obtained from a pool of *R. pulchellus*. A single isolate of KADV, the only African member of the tickborne flavivirus group, was recovered from a pool of *R. appendiculatus*. Six isolates were found by CF test to be indistinguishable from Barur virus, a rhabdovirus. Further characterization of these isolates was not conducted.

Three isolates of FMDV were identified by using panviral microarray-based technology. RNA isolated from viral culture of 1 isolate was reverse transcribed, randomly amplified, and hybridized to panviral DNA microarrays as described (*19*). Analysis of the hybridization patterns showed extensive hybridization to oligonucleotides derived from FMDV. Based on this result, PCR primers were designed from conserved regions of FMDV to confirm the identity of the virus. A PCR product of ≈600 bp was generated; it possessed 97% nucleotide identity to FMDV serotype SAT3. Two other isolates were identical by CF test.

Four isolates remained unidentified. Three of these were recovered from *R. pulchellus* and 1 from *A. gemma*. All of these isolates failed to react with the hyperimmune ascites grouping fluids used for the IFA identification procedure and produced negative results in RT-PCR tests when primers specific to known African tickborne viruses were used.

## Discussion

In this study, *A. gemma* ticks were incriminated for the first time as key vectors or reservoirs of tickborne viruses in the East African region; 46% of our virus isolates were obtained from this species. Distribution limits of ticks are variable and are influenced by several factors, including climate, vegetation, host density, host susceptibility, and host grazing habits. During previous studies conducted at the Lake Victoria basin in Kenya (*1*), *A. gemma* was not collected, most likely because this species is limited to more arid zones. *A. gemma* is found only in the dry zones of bushwillows (*Combretum*) and shrub steppe and is much more restricted than *A. lepidum* to very dry areas. DUGV and DUGV-like viruses were the most frequently isolated viruses in this study (33/56, 59%), and 64% of these isolates were from pools of *A. gemma*. Four of the 6 THOV isolates were obtained from pools of this species as well. Our results suggest that viruses are being actively transmitted in the drier parts of East Africa where *A. gemma* is more common. The pastoral regions, which supply many of the animals slaughtered at abattoirs in Nairobi, are predominantly dry and therefore likely to harbor this tick species in abundance.

The proportion of *R. appendiculatus* collected in this study was small when one considers the distribution of this tick in Kenya and its importance as a pest, a finding that suggests that most sampled cattle came from climatic zones where this species is not abundant. In Tanzania and Kenya, *R. appendiculatus* is most abundant in areas receiving >1,000 mm mean annual rainfall. It is absent from xerophytic and dry thicket zones with overgrazed pastures and little grass cover (*15*). NSDV is mainly transmitted by *R. appendiculatus*, and the virus is found only in areas where this species is abundant (*14*); therefore, the relatively low numbers of this species collected may explain why NSDV was not isolated in this study. Pools of *R. appendiculatus*, however, did yield single isolates of BHAV and KADV. BHAV has been isolated previously in Kenya and Nigeria (*1**,**20*). The medical implications of this virus for humans and animals in this region have not been determined, although the virus has been associated with human infection and illness in eastern Europe and West Africa (*21**–**23*). KADV is the only known African tickborne flavivirus. The virus was first isolated from *R. pravus* ticks taken from a cow in Uganda (*24**,**25*) and later in Kenya from *A. variegatum* and *R. pulchellus* (*14*). Although KADV pathogenicity is not evident in humans, antibodies against KADV were detected in human sera during a serosurvey in Uganda (*26*).

DUGV is commonly isolated in surveillance studies conducted in Africa (*1**,**2**,**27**,**28*), and it appears to be endemic in most of the drier parts of the continent. The implications of DUGV for human health have not been evaluated in Kenya, although reports from other regions in Africa suggest that human infection and illness caused by DUGV infection occur (*2**,**22**,**27*). Johnson et al. (*1*), in an earlier study conducted around Lake Victoria, recovered more DUGV isolates than any other virus and observed that more tick pools from dry scrub land (away from the lake) were infected with DUGV than pools from the swamp edge. These researchers also observed that 12 of the 39 DUGV isolates recovered varied in their behavior in cell culture and in suckling mice, which suggests that some of the DUGV strains isolated were different. In our study, in addition to the 26 isolates of DUGV, we identified 2 DUGV-like viruses (6 isolates of one, 1 isolate of another), which were found to differ significantly in S segment nucleotide sequence from previously published DUGV sequences. Further investigation of these isolates is necessary to determine their relatedness to DUGV.

THOV was first isolated in Kenya from *Rhipicephalus* species and *Boophilus decoloratus* in the 1930s (*29*) and has been isolated repeatedly from various tick species in Kenya, West Africa, Europe, and Asia (*30*). Two THOV infections have been reported in humans, with 1 fatality (*22*). In our survey, THOV was isolated from pools of *A. gemma* (*4*), *A. lepidum* (*1*), and *R. pulchellus* (*1*). DHOV, also a member of the *Thogotovirus* genus in the *Orthomyxovirdae* family, has been previously isolated in Europe, Asia, and the Middle East (*31**–**34*). Human DHOV infection has been evidenced by serologic survey results and human illness (*23**,**34**,**35*). We report here the first isolation of DHOV in East Africa. This finding suggests a southward spread of the virus that is supported by the presence of competent tick vectors in the region and demonstrates the potential for other tickborne viruses circulating in Europe and Asia to spread to the African continent. Such spread would have adverse consequences for large, immunologically naive populations whose pastoral practices provide for closer human-animal contact.

An unexpected finding in this study was the isolation of FMDV from 3 pools of *R. pulchellus*. FMDV is endemic in many parts of Africa; however, it has not previously been identified in association with tick surveillance or transmission studies (*36*). This finding does not constitute evidence that FMDV replicates in or can be transmitted by ticks; in fact, previous reports indicate that the virus is not transmissible by *Rhipicephalus* ticks or blood-feeding flies (*37**,**38*). However, the virus has been demonstrated to persist in ticks for up to several days after feeding on an infected animal (*37*). The ticks in our study were not held for any length of time to allow for blood in the ticks to be digested before processing for virus isolation. Therefore, FMDV may have been present in undigested blood in >1 ticks in each pool. FMDV is present in the blood of an infected animal, skin lesions, and skin areas that do not contain lesions (*39*). The virus persists in skin up to 4 days beyond the period of viremia and for extended periods in preserved hides (*40*). Therefore, mouth parts of ticks feeding on FMDV-infected cattle might have become contaminated with the virus, which was then not sufficiently exposed to the external rinsing procedures to which the ticks were subjected before processing. Further investigation is necessary to clarify the mechanism of these FMDV isolations and the implications of these findings.

Our study illustrates the potential for tickborne dissemination of endemic and emergent viruses, some of which are human pathogens, among livestock as well as the potential for transmission of these pathogens to humans. Regular surveillance is warranted to monitor the presence and spread of these and other viruses facilitated through livestock rearing, marketing, and movement in Africa.
